# Supplementary figures and images for: Genome-Wide Association Mapping of Correlated Traits in Cassava: Dry Matter and Total Carotenoid Content
Source: Plant Genome. 2017 Aug 3;10(3):10.3835/plantgenome2016.09.0094. doi: 10.3835/plantgenome2016.09.0094 (PMC7822061; doi:10.3835/plantgenome2016.09.0094)

1.

2.

3.

4.

5.

6.

7.

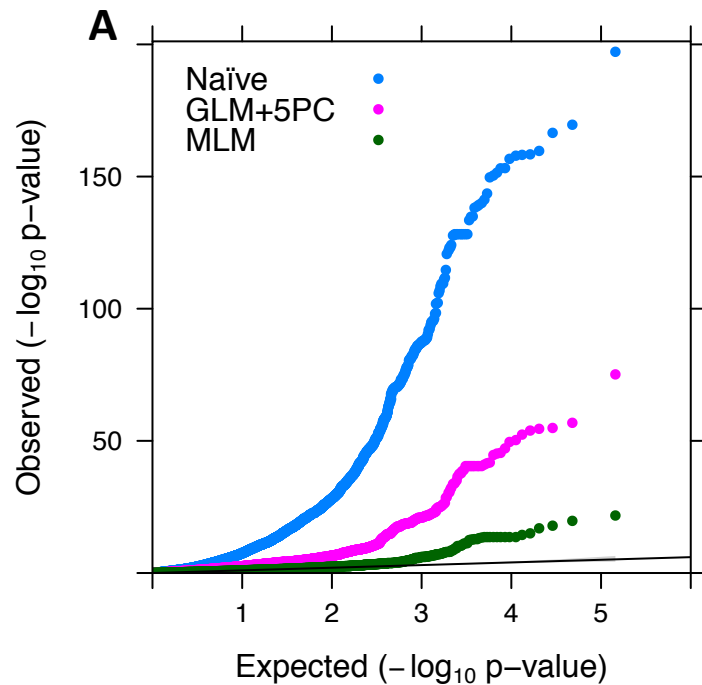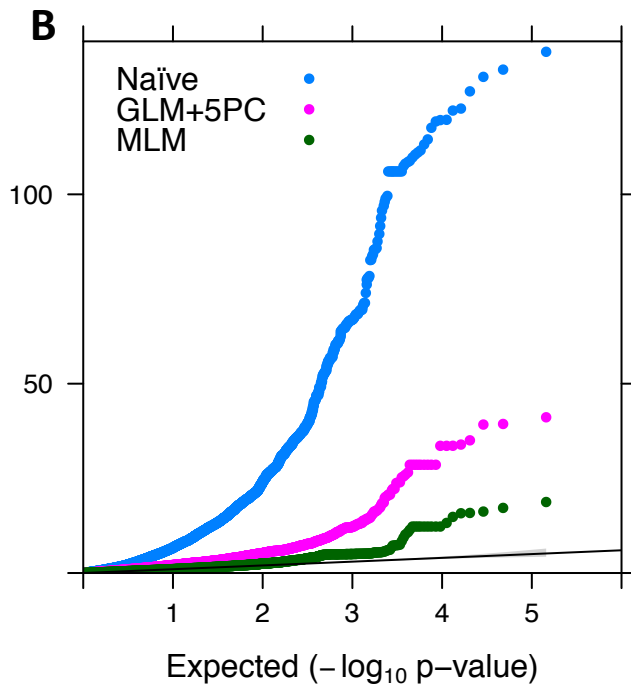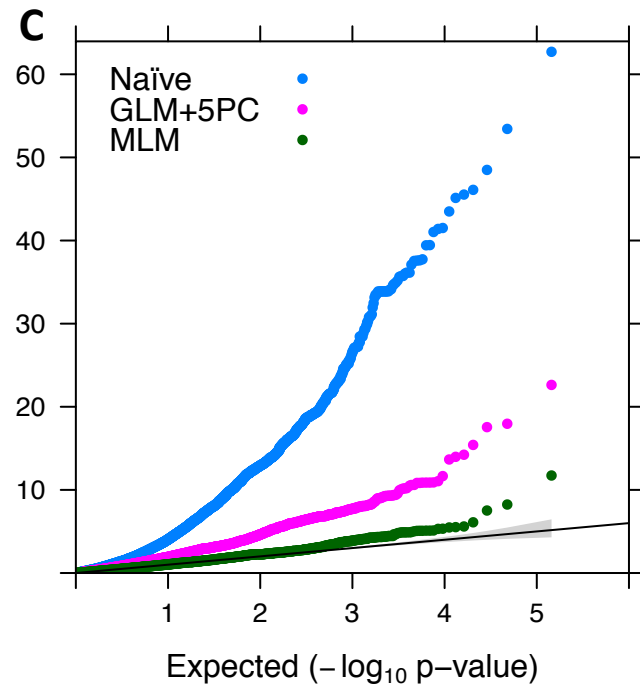

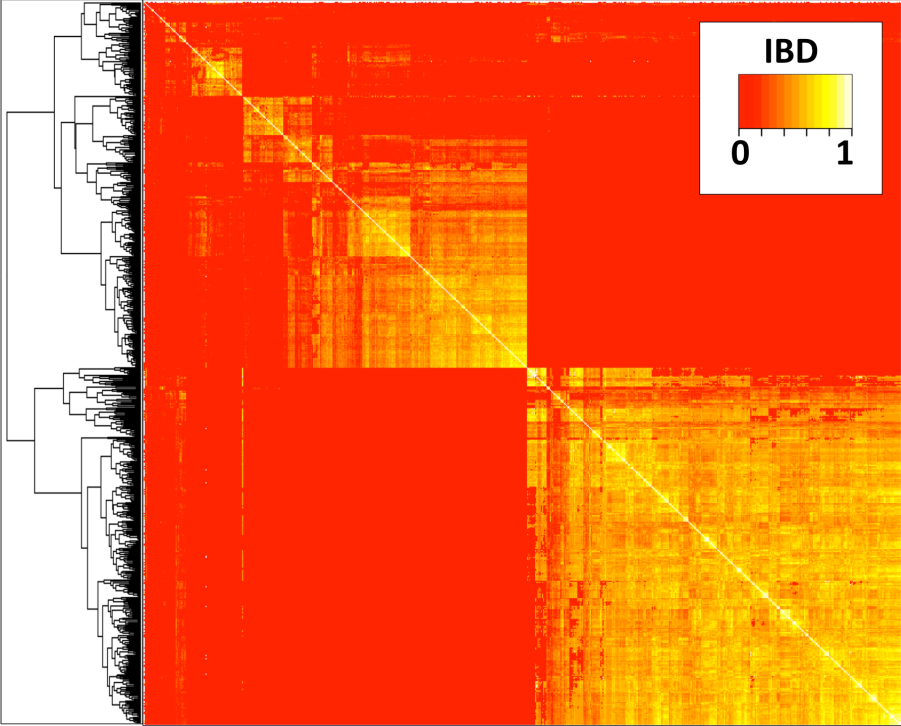

Supplement: Supplementary file 1 [file TPG-10-03-0094-s001.pdf]
